# Unraveling the Heterogeneity of Sarcoma Survivors’ Health-Related Quality of Life Regarding Primary Sarcoma Location: Results from the SURVSARC Study

**DOI:** 10.3390/cancers12113083

**Published:** 2020-10-22

**Authors:** Ilse van Eck, Dide den Hollander, Ingrid M.E. Desar, Vicky L.M.N. Soomers, Michiel A.J. van de Sande, Jacco J. de Haan, Cornelis Verhoef, Ingeborg J.H. Vriens, Johannes J. Bonenkamp, Winette T.A. van der Graaf, Winan J. van Houdt, Olga Husson

**Affiliations:** 1 Department of Medical Oncology, The Netherlands Cancer Institute, Antoni van Leeuwenhoek, 1066 CX Amsterdam, The Netherlands; i.vaneck@student.ru.nl (I.v.E.); D.denhollander@radboudumc.nl (D.d.H.); w.vd.graaf@nki.nl (W.T.A.v.d.G.); 2 Department of Medical Oncology, Radboud University Medical Center, 6525 GA Nijmegen, The Netherlands; ingrid.desar@radboudumc.nl (I.M.E.D.); vicky.soomers@radboudumc.nl (V.L.M.N.S.); 3 Department of Orthopedics, Leiden University Medical Center, 2333 ZA Leiden, The Netherlands; m.a.j.van_de_sande@lumc.nl; 4 Department of Medical Oncology, University Medical Center Groningen, 9713 GZ Groningen, The Netherlands; j.j.de.haan@umcg.nl; 5 Department of Surgical Oncology, Erasmus MC Cancer Institute, 3015 GD Rotterdam, The Netherlands; c.verhoef@erasmusmc.nl; 6 Department of Medical Oncology, Maastricht University Medical Center, 6229 HX Maastricht, The Netherlands; ingeborg.vriens@mumc.nl; 7 Department of Surgical Oncology, Radboud University Medical Center, 6525 GA Nijmegen, The Netherlands; han.bonenkamp@radboudumc.nl; 8 Department of Surgical Oncology, The Netherlands Cancer Institute, Antoni van Leeuwenhoek, 1066 CX Amsterdam, The Netherlands; w.v.houdt@nki.nl; 9 Division of Clinical Studies, Institute of Cancer Research, London SM2 5NG, UK;

**Keywords:** sarcomas, soft tissue sarcoma, bone sarcoma, health-related quality of life, patient-reported outcomes

## Abstract

**Simple Summary:**

Sarcomas are a rare group of heterogenous tumors that can develop anywhere in the body. Currently, studies on health-related quality of life (HRQoL) focus on sarcomas of the arm and leg or have too small sample sizes to examine the heterogeneity between different sarcoma locations, leading to limited insight into HRQoL of survivors with specific sarcoma locations. The aim of this study was to assess differences in HRQoL and examine treatment-specific HRQoL issues per sarcoma location. We found, in a population of 1099 sarcoma survivors, different patterns of HRQoL according to primary sarcoma location and a high number of additional, unique treatment-specific HRQoL issues per location, which were not captured with the general HRQoL questionnaire used in cancer patients. This indicates that the currently used HRQoL measures are too generic to capture all sarcoma-related issues, emphasizing the necessity for a comprehensive sarcoma-specific HRQoL measurement strategy.

**Abstract:**

Sarcoma patients experience physical and psychological symptoms, depending on age of onset, subtype, treatment, stage, and location of the sarcoma, which can adversely affect patients’ health-related quality of life (HRQoL). This study aimed to unravel the heterogeneity of sarcoma survivors’ HRQoL regarding primary sarcoma location. A cross-sectional study was conducted among Dutch sarcoma survivors (*N* = 1099) aged ≥18, diagnosed 2–10 years ago. Primary sarcoma locations were head and neck, chest, abdominal including retroperitoneal, pelvis including urogenital organs, axial skeleton, extremities (upper and lower), breast, skin and other locations. The European Organization for Research and Treatment of Cancer—Quality of Life Questionnaire (EORTC QLQ)-C30 was used to measure HRQoL accompanied by treatment-specific HRQoL questions. Sociodemographic and clinical characteristics were collected from the Netherlands Cancer Registry. Axial skeleton sarcomas had the lowest functioning levels and highest symptoms compared to other locations. Skin sarcomas had the highest functioning levels and lowest symptoms on most scales. Bone sarcomas scored worse on several HRQoL domains compared to soft tissue sarcomas. High prevalence of treatment-specific HRQoL issues were found per location. In conclusion, sarcomas can present everywhere, which is reflected by different HRQoL outcomes according to primary sarcoma location. The currently used HRQoL measure lacks treatment-specific questions and is too generic to capture all sarcoma-related issues, emphasizing the necessity for a comprehensive sarcoma-specific HRQoL measurement strategy.

## 1. Introduction

Sarcomas are a group of heterogeneous neoplasms of mesenchymal origin, covering more than 100 histological subtypes with differences in stage at diagnosis, prognosis, and treatment [1]. They can develop at any age and at any anatomical site in the body [2]. Sarcomas are rare cancers and account for less than 1% of all adult solid cancers [2]. They are broadly divided into soft tissue sarcomas (STS) including gastrointestinal stromal tumors (GIST) ( 84%), bone sarcomas (BS: 14%) or other sarcomas [3]. The overall incidence of sarcomas in Europe is about 7 in 100,000 persons, with 30,000 new cases a year [3]. The five-year relative survival in Europe (period 2000–2007) is 60% for STS and 50% for BS [4].

The first choice of treatment for localized STS is surgery combined with (neo)adjuvant radiotherapy (RT) and/or (neo)adjuvant chemotherapy, dependent on patient characteristics, tumor grade, histology, and location of the tumor [5]. For patients with BS, the preferred treatment modality is surgery as well, combined with chemotherapy in case of osteosarcoma and a combination of chemotherapy and RT in case of Ewing’s sarcoma [6]. However, not all sarcomas are amenable to surgical resection due to their anatomical location. For specific subtypes, such as (para) spinal sarcomas, proton therapy can be considered in order to minimize delivery of high-dose RT to the surrounding tissues [7]. Furthermore, in patients with non-resectable extremity STS, isolated limb perfusion (ILP) using melphalan and tumor necrosis factor alpha can achieve limb salvage [8]. Un-resectable metastatic sarcomas are often treated with palliative chemotherapy and/or RT [6,9].

All these different treatments are mainly focused on tumor reduction and improvement of survival. Treatment is often accompanied by significant short- and long-term side effects that negatively affect patients’ functioning in daily life and consequently limit the overall advantages of therapy [10]. Research performed in cancer patients in general has shown an increasing need to not only focus on survival rates, but also on patient reported outcomes such as health-related quality of life (HRQoL) [11]. HRQoL is a multidimensional concept that includes the patient’s perception of the impact of the disease and its treatment on domains of physical-, role-, social-, emotional- and cognitive-functioning [10].

There are generally few studies that investigate HRQoL of sarcoma patients [12,13,14]. In addition, the available studies often have small sample sizes that do not allow examination of the entire collective of sarcoma patients for heterogeneity at all. The small number of studies with larger sample sizes often focus on the sarcoma patient population as a whole or only on patients with extremity sarcomas and often do not include other important sarcoma locations, leading to limited insight into differences between subgroups of patients. Considering that sarcomas can develop anywhere in the body (epidemiology in the Netherlands BS; lower extremities: 47%, upper extremities: 23%, torso: 23%, head and neck: 7% and STS: lower extremities: 30%, upper extremities: 12%, torso: 32%, head and neck: 15%, retroperitoneal/mediastinum/heart: 9% [15]) and have different treatment strategies with different potential short- and long-term adverse events, it is difficult to use these results to provide personalized care for patients with specific sarcoma locations. A few studies have tried to overcome this by focusing on one location. For example, patients with skull base chordomas showed lower mental HRQoL scores compared to the general population, due to neurological deficits, pain medication use, and higher levels of depression [16]. In contrast, extremity sarcoma patients who had an amputation scored lower on the physical functional scale than those whose limbs were spared [17]. In addition, literature shows that for patients treated for extremity STS, restrictions in participation of life roles had the greatest impact on patients’ HRQoL [18].

The objectives of this study are (1) assessing the differences in HRQoL between survivors with different primary sarcoma tumor locations (head and neck, chest, abdominal including retroperitoneal, pelvis including urogenital organs, axial skeleton, upper and lower extremities, breast, skin, and other locations); (2) exploratively examining of treatment-specific HRQoL issues per sarcoma location. The results of this study can be used to understand specific problems and needs of survivors with different locations of the tumor, improve personalized (after)care for sarcoma survivors tailored to a specific sarcoma location, and optimize shared decision-making.

## 2. Results

### 2.1. Patient Characteristics

A total of 1099 (response rate 58%) survivors were included with a mean age of 60 years (range 18–90). The majority of survivors were diagnosed with STS (75%), had stage I (44%) or stage II (29%) sarcoma, and underwent surgery (41%) or surgery in combination with RT (38%). Mean time since diagnosis was 67 ± 30 months. Most survivors were married, had civil partnerships or cohabited (78%), went to college or university (77%), and worked full- or part-time (50%). Sarcoma locations were categorized into 10 categories, with most survivors in the lower extremity sarcoma group (*n* = 405), and the least survivors in the breast sarcoma group (*n* = 25). Gender, sarcoma subtype, tumor stage, treatment, comorbidities, and marital status were significantly different between sarcoma locations. The other patient characteristics were similar between the location groups (Table 1). The non-responder analyses have been published before, indicating a younger age at diagnosis and at study enrolment, and a lower social-economic status (SES) for non-responders [19].

### 2.2. Mean HRQoL Scores for Each Domain of the EORTC-QLQ-C30

**Figure 1:**
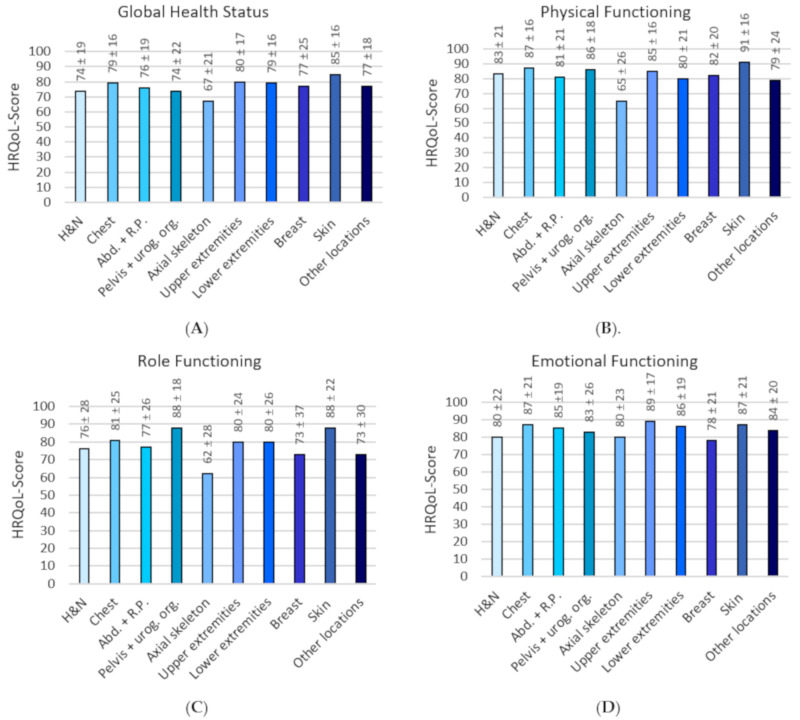

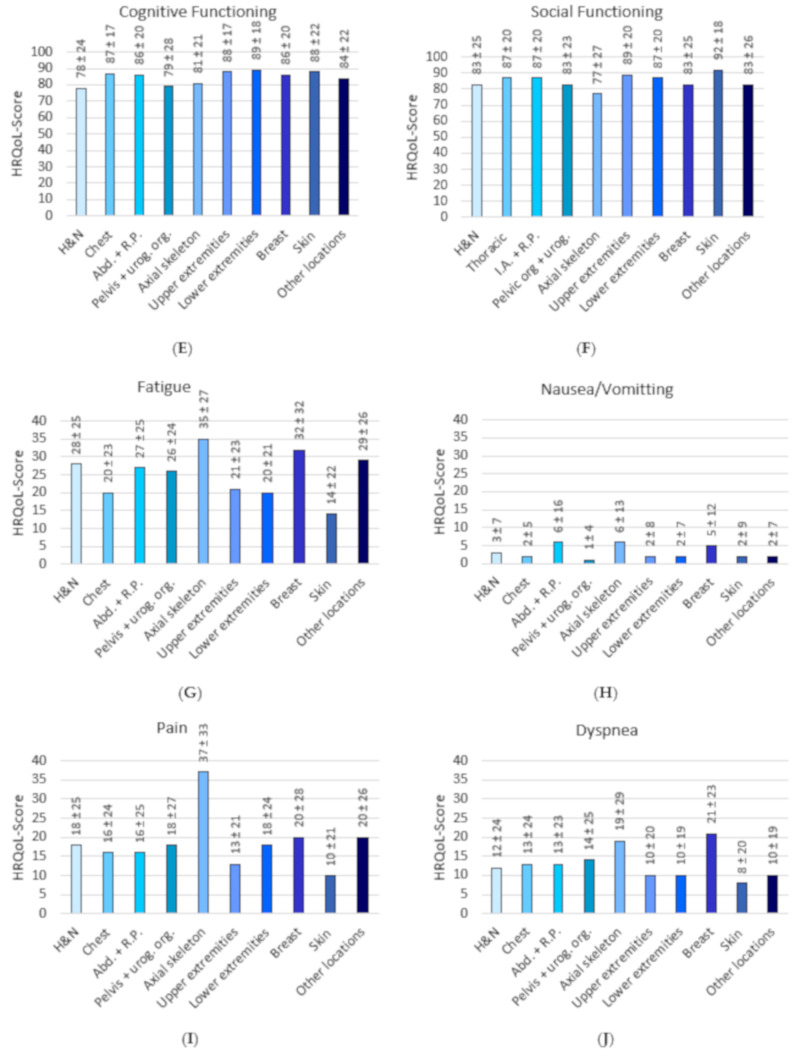

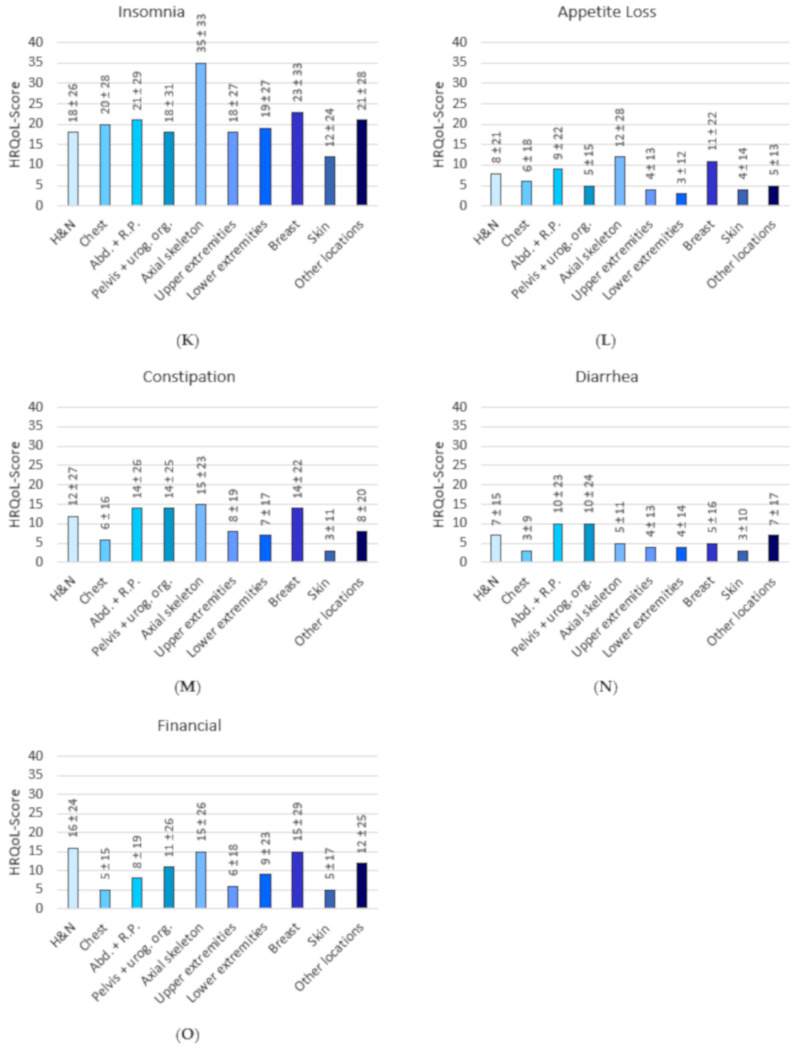
Mean health-related quality of life (HRQoL) scores on the EORTC-QLQ-C30. (**A–O**): Mean HRQoL score per location for all domains of HRQoL. * H and N = Head and Neck, Abd. and R.P. = Abdominal including retroperitoneal, Pelvis and urog. org. = pelvis including urogenital organs.

Mean HRQoL scores for the EORTC-QLQ-C30 are presented in Figure 1 (A–O). Of all sarcoma locations, survivors with sarcoma of the axial skeleton had the lowest mean scores on all functioning scales except for emotional (lowest for breast sarcomas) and cognitive functioning (lowest for head and neck sarcomas) and the highest scores on the symptom scales and single items, except for nausea/vomiting (equal with axial skeleton, for abdominal including retroperitoneal sarcomas), dyspnea (highest in breast sarcomas), diarrhea (highest in abdominal including retroperitoneal sarcomas and pelvis including urogenital organ sarcomas) and financial impact (highest in head and neck sarcomas). Survivors with skin sarcomas had the highest scores on global health status, physical functioning, and social functioning and equal on role functioning with pelvis including urogenital organ sarcomas. Furthermore, skin sarcoma survivors had the lowest scores on the symptom scales and single items, except for nausea/vomiting (lowest in pelvis including urogenital organ sarcomas), appetite loss (lowest in lower extremity sarcomas), diarrhea and financial problems (both lowest in chest sarcomas).

### 2.3. Influence of Location on HRQoL

Unadjusted and adjusted Mean Differences (MD) on the HRQoL domains are presented in Table 2 and Table S1. After adjustment for confounders, axial skeleton sarcoma survivors still reported significantly lower global health status and lower role functioning compared to chest, upper and lower extremities, and skin sarcoma survivors. MDs varied from 10.1 to 14.2 for global health status (medium clinical relevance) and from 9.2 to 12.8 for role functioning (small clinical relevance). Physical functioning was lower in axial skeleton sarcoma survivors compared to all remaining sarcoma locations, except for ‘other location’ sarcomas. The MDs varied from 8.9 for lower extremity sarcomas (small clinical relevance) to 18.9 for chest sarcomas (median clinical relevance). Social functioning was lower in axial skeleton sarcoma survivors compared to survivors with upper (MD = 8.7) and lower extremity sarcomas (MD = 7.0), both were of small clinical relevance. For emotional and cognitive functioning no significant differences were seen between axial skeleton sarcomas and all remaining locations (Table 2).

After adjustment for confounders, the majority of symptoms were still worse in axial skeleton sarcoma survivors. Axial skeleton sarcoma survivors reported more fatigue compared to chest (MD = −12.0), upper (MD = −10.1) and lower extremity (MD = −9.7), and skin sarcoma survivors (MD = −10.5); all MDs were of small clinical relevance. Pain was worse in axial skeleton survivors compared to all remaining locations except for breast sarcomas. MDs varied from −12.4 for pelvis including urogenital organ sarcomas (small clinical difference) to −21.5 for upper extremity sarcomas (large clinical relevance). Furthermore, axial skeleton sarcoma survivors had higher levels of insomnia compared to upper (MD = −15.5) and lower extremity (MD = −13.9) and skin sarcomas (MD = −17.7) and more appetite loss compared to lower extremity sarcomas (MD = −8.6). The MDs for insomnia were of medium clinical relevance, whereas the MDs for appetite loss were of small clinical relevance. No significant differences between axial skeleton sarcomas and other locations were seen for nausea and vomiting, dyspnea, constipation, diarrhea, and financial consequences (Table 2).

Abdominal including retroperitoneal sarcoma survivors suffered most from gastro-intestinal complaints. More nausea and vomiting were seen compared to chest and upper and lower extremity sarcomas, more appetite loss was seen compared to lower extremity sarcomas, more constipation compared to skin sarcomas and more diarrhea compared to chest, lower extremity, and skin sarcomas. All differences were of small clinical relevance except the MD for diarrhea between abdominal and chest sarcoma survivors, which was of medium clinical relevance (Table S1).

Skin sarcoma survivors reported better global health status compared to head and neck and abdominal including retroperitoneal sarcoma survivors, better physical functioning compared to lower extremity and ‘other location’ sarcomas, better role functioning compared to ‘other location’ sarcomas, and less fatigue compared to breast and ‘other location’ sarcomas. All MDs were of small clinical relevance (Table S1).

Lastly, head and neck sarcoma survivors reported worse cognitive functioning scores, of small clinical relevance, compared to upper and lower extremity sarcoma survivors (Table S1).

BS survivors scored worse on several HRQoL domains compared to STS survivors. Worse social functioning (MD: −12.8, *p* = 0.036) was seen for BS of the head and neck, higher financial impact (MD: 8.9, *p* = 0.027) was seen for BS of the upper extremities and worse physical (MD: −11.9, *p* < 0.000) and role functioning (MD: −14.3, *p* < 0.000) was seen for BS of the lower extremities. The MDs were all of small clinical relevance, except for social functioning, which was of medium clinical relevance. No differences in HRQoL scores were seen between BS and STS of the chest.

### 2.4. Treatment-Specific HRQoL Issues per Sarcoma Location

Only the presence of treatment-specific HRQoL issues per sarcoma location with an occurrence of ≥20% are mentioned. For an overview of all treatment-specific HRQoL issues per sarcoma location, see Tables S2–S8 in the Supplementary Materials.

Upper extremity sarcoma survivors (response rate (RR): 71%) reported the following activities more frequently as difficult after surgery than not difficult: performing heavy household chores (39%), lifting a box to an overhead shelf (37%), gardening (32%), and participating in usual sporting activities (32%). Other common activities reported as difficult were carrying a shopping bag (28%), completing usual duties at work/at home (27%), participating in usual leisure activities (26%), cutting food while eating (23%), giving or receiving change (22%), preparing and serving meals (22%), and tying shoe laces (20%). Of the 17 upper extremity survivors who underwent an amputation (21%), eight (47%) suffered from phantom pain.

Lower extremity sarcoma survivors (RR: 87%) reported the following activities more frequently as difficult after surgery than not difficult: getting up from kneeling (57%), kneeling down (56%), walking up or down a hill or a ramp (46%), and participating in usual sporting activities (45%). Other common activities reported as difficult were performing heavy household chores (39%), bending to pick something up off the floor (37%), gardening (37%), walking up- (37%) and downstairs (34%), getting in and out of a car (34%), participating in usual leisure activities (34%), completing usual duties at work/at home (33%), walking outdoors (31%), shopping (31%), putting on a pair of socks or stockings (30%), shoes (28%) or trousers (25%), rising from a chair (27%), working the usual number of hours (26%), participating in sexual activities (23%), and getting in and out of the bath (22%). Of the 37 lower extremity survivors who underwent an amputation (10%), 25 (68%) reported phantom pain. Furthermore, in both upper and lower extremity sarcoma survivors, survivors with an amputation more often reported task difficulties compared to survivors without an amputation.

The most common symptoms reported after surgery by abdominal including retroperitoneal sarcoma survivors (RR: 85%) were worries about weight (36%), abdominal pain (32%), a bloated feeling in the abdomen (32%), and a feeling of fullness too quickly while eating (22%). The seven abdominal survivors who had an ostomy after surgery (8%) also reported unintentional release of gas/flatulence from the stoma bag (43%), feeling embarrassed due to the ostomy (43%), and leakage of stool from the stoma bag (29%) as common issues.

Frequent reported issues after surgery by head and neck sarcoma survivors (RR: 71%) were problems with clearly speaking (35%), a dry mouth (33%), problems with smell (28%), chewing problems (25%), problems with sense of taste (24%), pain in the operated area (22%), trouble eating in front of other people (22%), and a sensitive mouth (21%).

Breast sarcoma survivors (RR: 80%), of whom 5 (20%) had breast reconstruction after surgery, reported difficulties looking at themselves naked (48%), difficulties raising the arm or moving it sideways (32%), and pain in the area of the affected breast (28%) after surgery.

Cervix, uterus, and ovary (RR 88%) sarcoma survivors reported pain in the back (53%), flatulence (53%), complaints similar to the menopause (53%), abdominal cramps (41%), a bloated feeling in the abdomen (41%), a need to hurry to the toilet if they felt the urge to pass urine (41%), and unintentional release of urine (35%) as common symptoms after surgery.

Common symptoms reported after chemotherapy (n = 86) were tingling or numbness in hands or feet (43%), skin problems (41%), pain in muscles or joints (38%), problems with hair (34%), a dry mouth (34%), different taste (22%), and a sensitive mouth (22%). Common symptoms reported after RT (*n* = 426) were muscle weakness or loss of sensation at site of radiation (47%), scars at site of radiation (45%), skin problems (35%), and muscle cramps at site of radiation (22%). Lastly, common symptoms reported after chemoradiation (*n* = 98) were a dry mouth (45%), muscle weakness or loss of sensation at site of radiation (44%), skin problems (43%), tingling or numbness in hands or feet (43%), different taste (42%), problems with hair (41%), pain in muscles or joints (40%), scars at site of radiation (31%), and a sensitive mouth (30%).

## 3. Discussion

This population-based cross-sectional study, which aimed to unravel the heterogeneity of HRQoL in sarcoma survivors according to primary sarcoma location, revealed significantly worse HRQoL outcomes for axial skeleton sarcomas on most domains of the EORTC-QLQ-C30 compared to other locations. The highest HRQoL outcomes were seen for skin sarcomas, however, after adjustment for confounders, only a number of domains of HRQoL remained significant compared to other locations. Abdominal including retroperitoneal sarcoma survivors had the worst scores on gastro-intestinal symptoms compared to the other locations, whereas head and neck sarcoma survivors scored worse on cognitive functioning. For the remaining sarcoma locations, no significant differences in HRQoL outcomes between the locations were found. BS survivors scored worse on several HRQoL domains compared to STS survivors. The analysis of treatment-specific HRQoL issues per sarcoma location revealed high levels of functional impairment and side-effects or complaints which differed per location and were unique for the specific treatment strategies of a specific sarcoma location.

Previous research mainly focused on HRQoL in patients with extremity sarcomas or has too small sample sizes to allow examination of the entire collective of sarcoma patients for heterogeneity, making it challenging to compare the results to the existing literature. Nevertheless, the study of Hoffmann et al. [21], also using the EORTC-QLQ-C30 to measure HRQoL, showed deficiencies in global health status, role-, social-, and physical functioning for pelvic sarcomas compared to the normal population, whereas no substantial deficiencies in emotional functioning were seen. Even though these results were compared to the general population, the significant lower scores on these domains of HRQoL are in line with our findings comparing axial skeleton (spine and pelvic bones) to other locations. Moreover, higher pain scores for spine sarcomas and more pronounced pain-related restrictions in the activities of daily living for pelvic sarcomas compared to the normative population are described [21,22].

So far, no studies compared HRQoL of skin sarcomas to other locations or to the general population. The skin sarcoma survivors in our study were mainly diagnosed with dermatofibrosarcoma protuberans (66%), a histological subtype that has a good prognosis in terms of survival if treated adequately and rarely requires RT or systemic therapy [23,24]. Treatment and prognosis of this type of skin sarcoma is comparable to treatment and prognosis of non-melanoma skin cancer survivors. In the latter group, high HRQoL scores similar to those of the healthy population were described [25], which is comparable to our skin sarcoma population.

The high levels of gastro-intestinal complaints in abdominal including retroperitoneal sarcomas are not unexpected and are in line with the literature. The study of Wong et al. [26], investigating HRQoL using the EORTC QLQ-C30 in retroperitoneal sarcomas treated with pre-operative RT and surgery, showed a mean HRQoL improvement at one-month post-RT, whereas the diarrhea symptom scale worsened from baseline. There were fifty-four percent of patients with gastrointestinal toxicities by the end of RT and 19% had chronic RT- and/or surgery-related gastro-intestinal toxicities at 36 months post-RT [26]. The study of Callegaro et al. [27] mentioned changes in bowel habits after surgery in retroperitoneal sarcoma patients as well, which involved constipation, diarrhea, or alternating symptoms of diarrhea and constipation.

Finally, head and neck sarcoma survivors had worse cognitive functioning, a finding in line with the known literature showing that fatigue, sleep disturbances, and cognitive impairments are prevalent and clinically important problems among head and neck cancer patients [28]. High fatigue scores were also seen in our head and neck sarcoma population, but were not significantly different from other locations.

For the remaining locations, we found no significant differences in HRQoL domains compared to each other. In contrast to the generic HRQoL questionnaire, the analysis of the questions about treatment-specific HRQoL issues per sarcoma location showed high levels of functional impairment and side effects that differed per location and were unique for specific treatment strategies of a specific sarcoma location. A symptom that was common for all kinds of sarcoma locations was pain after surgery. Sarcoma patients are at risk for many kinds of pain [29] and although information on pain medication was lacking in this study, this indicates that adequate pain control is important for maintaining function and HRQoL after sarcoma treatment. In addition, both upper and lower extremity sarcoma survivors experienced a lot of functional difficulties and restrictions in work, leisure activities, and sports after surgery. The study of Schreiber et al. [18] showed that restriction in participation of life roles and situations had the greatest effect on extremity sarcoma patients’ HRQOL, suggesting that sarcoma patients should be supported to re-integrate into normal work/life after treatment. Another remarkable result found in this study is the high percentage of hair problems reported after completing chemotherapy (34%) or chemoradiation (41%), whereas it was expected that this would have been recovered 2–10 years after ending chemotherapy. Nevertheless, Kang et al. [30] found that in breast cancer patients, 42.3% of the patients still suffer from permanent chemotherapy-induced alopecia at 3 years follow-up. Hair thinning was the most common problem reported by the breast cancer survivors (75.0%), followed by reduced hair volume (53.9%), hair loss (34.6%), and grey hair (34.6%) [30]. Hair problems can affect a person’s self-image, therefore clinicians should be aware of this distressing long-term issue and should properly inform patients about this side effect or about prevention strategies such as scalp cooling [30,31]. Additionally for the other locations, treatment-specific issues were found which require attention, for example, difficulties seeing themselves naked for breast sarcomas, urine incontinence for uterus sarcomas, or speaking problems for head and neck sarcomas, issues that will probably have an impact on the survivors’ HRQoL.

A substantial part of the issues assessed with the treatment-specific HRQoL questions were not captured by the EORTC-QLQ-C30, which might indicate that, due to the heterogeneity of the sarcoma population, the current EORTC-QLQ-C30 questionnaire is not able to adequately assess all relevant HRQoL issues. Recently, a new patient-reported experience questionnaire for patients with sarcoma was designed by Martins et al. [32], the Sarcoma Assessment Measure (SAM). Nevertheless, whereas the SAM is suitable to assess the sarcoma patients’ experience to delivered care according to their needs, Husson et al. [33] suggested that it is not the best measurement strategy for incorporating the sarcoma patients’ voice in clinical research and capturing the heterogeneity of sarcomas. Therefore, a more flexible measurement approach is recommended, for example, combining a standardized generic or cancer-specific HRQoL measure with validated items from Item Libraries [33]. The EORTC Quality of Life Group (QLG) and the EORTC Soft Tissue and Bone Sarcoma Group (STBSG) are currently investigating an international (global) measurement strategy for sarcoma patients using the EORTC QLQ-C30 in conjunction with flexible item lists from the EORTC Item Library (ClinicalTrials.gov Identifier: NCT04071704).

So far, many studies investigating HRQoL in sarcoma patients focused on extremity sarcoma patients or had a too small sample sizes to allow examination of the entire collective of sarcoma patients for heterogeneity. By unraveling the heterogeneity in HRQoL of sarcoma survivors per tumor location, results of this study improve the applicability of patient-reported outcomes to specific sarcoma subgroups. Because this study also investigated specific HRQoL issues associated with specific treatments of the various sarcoma locations, results can be used to improve personalized supportive care for sarcoma survivors tailored to a specific sarcoma location. Other strengths of this study are the large study population and the population-based character.

This study also has some limitations. Even though this study describes a large study population, some specific sarcoma subgroups (breast and pelvis including urogenital organ sarcomas) consisted of few participants, resulting in limitations of statistical power. The small number of urogenital sarcomas might be explained by the fact that these patients are regularly, but not always, treated in reference centers. Secondly, all included survivors were diagnosed 2–10 years after diagnoses, therefore the reported complaints might be attributable to other causes, as the diagnoses and treatment of the sarcoma took place a long time ago. In addition, it can also lead to survivorship bias, as our study only included the survivors who survived the disease and probably have a better HRQoL than those with metastatic sarcomas [34] (e.g., the 5-year relative survival of high-grade STS is 46%. In addition, 50% of the patients with metastatic STS at diagnosis do not undergo treatment and even have a shorter survival rate, these patients are therefore not presented in our study sample) [15]. Thirdly, we only included survivors diagnosed in third-line sarcoma expertise centers, comprising 51% of the total Dutch sarcoma population [15]. As a result, our study population is not fully representative of the entire sarcoma patient population. In addition, the majority of survivors were well educated, not fully representing the normal population. Fourthly, because we did not follow up with the patient over time, we have no information on whether the patient was still disease-free or received treatment for relapsed or metastatic disease while completing the questionnaire, influencing the patients HRQoL and experienced treatment side effects. Finally, because we analyzed the data from an existing dataset using a prior determined questionnaire with a focus on treatment-specific issues, we did not have information about treatment-specific HRQoL issues of all locations (i.e., axial skeleton and skin sarcomas).

## 4. Materials and Methods

### 4.1. Study Design and Participants

This population-based cross-sectional study recruited sarcoma survivors aged ≥18 years who are registered at the Netherlands Cancer Registry (NCR). The NCR collects patient and tumor characteristics of all newly diagnosed individuals with cancer in the Netherlands [35]. Only survivors who were diagnosed with sarcoma, independent of the intent of primary treatment, (according to the ICD-10-GM codes C40 and C41 for bone sarcoma and C49 for soft-tissue sarcoma [36]) between 1 April 2008 and 31 December 2016 at one of the six participating sarcoma expertise centers (Radboudumc Nijmegen, The Netherlands Cancer Institute Amsterdam, University Medical Centre Groningen, Leiden University Medical Centre, Erasmus MC Cancer Institute Rotterdam, Maastricht University Medical Centre) were included. Because all patients were diagnosed 2–10 years ago, we defined them as survivors in this study. Exclusion criteria were cognitive impairment, a physical condition that affected their ability to participate [u12] [i.13] (judged by their (ex-) treating physician), unverifiable address, or inability to read and write Dutch. Because of the indolent clinical behavior and less aggressive treatment strategies, patients with desmoid fibromatosis, grade I chondrosarcoma, atypical lipomatous tumors or giant-cell tumors were excluded. In addition, patients with GIST were excluded because of participation in another Dutch questionnaire study. Ethical approval was provided by the medical ethical committee of the Radboud University Medical Centre (2017-3944). According to the Dutch law, approval of one ethical committee for questionnaire research is valid for all participating centers. The study was registered in the Dutch Trial Registry (NTR-7253).

### 4.2. Recruitment and Data Collection

Sarcoma survivors who met the inclusion criteria were invited by a letter from their (former) treating healthcare professional. If written informed consent was provided, and the patient agreed to linkage of questionnaire data with their clinical data in the NCR, they received a login code to fill in the digital questionnaire. The login codes were not directly linked to the patient. On request the questionnaire could also be filled in by pencil and paper and was digitalized afterwards. Data collection was performed by employees of the Netherlands Comprehensive Cancer Organisation (IKNL) from October 2018 till June 2019 within PROFILES (Patient Reported Outcomes Following Initial treatment and Long-term Evaluation of Survivorship; www.profilesregistry.nl ) [37]. PROFILES is a protected data management system to study the physical and psychosocial impact of cancer and its treatment. Further details of the data collection method have been described previously [38].

### 4.3. Socio-Demographic and Clinical Characteristics

Socio-demographic and clinical characteristics (gender, age, tumor subtype, tumor grade, stage at diagnosis, tumor-location, primary treatment, and time since diagnosis) were derived from the NCR. Primary treatments received are described in the NCR as initial treatment after diagnosis.

Questions on marital status and education level were added to the questionnaire package. Comorbidity was assessed by the self-administered comorbidity questionnaire (SCQ) [39]. Tumor location was identified using the ICD-10 codes. Further categorization of the ICD-10 codes in location subgroups was based on clinical experience of sarcoma specialists, taking into account common problems and side effects for different anatomical locations. The different locations of the primary sarcoma were: head and neck, chest, abdominal including retroperitoneal, pelvis including urogenital organs, axial skeleton (spine and pelvic bones), upper and lower extremities, breast, skin, and other locations. For an overview of the specific sarcomas per location category, see Text S1.

### 4.4. HRQoL

HRQoL was assessed with the European Organization for Research and Treatment of Cancer—Quality of Life Questionnaire (EORTC QLQ)-C30. This 30-item validated questionnaire incorporates nine-multi-item scales: five functional scales (physical, role, cognitive, emotional, and social), three symptom scales (fatigue, pain and nausea, and vomiting), a global health and quality of life scale and a number of single items assessing common symptoms (dyspnea, loss of appetite, sleep disturbance, constipation and diarrhea) and perceived financial impact of the disease [40]. All items were scored on a 4-point Likert scale ranging from “not at all” to “very much”, except for the items regarding global health and quality of life which were scored from 1 (very poor) to 7 (excellent). All scores were linearly transformed to a 1–100 scale. Higher scores on global health, quality of life, physical-, role-, cognitive, emotional- and social scales represent better functioning, whereas a higher score on the symptom scales corresponds to more complaints.

### 4.5. Treatment-Specific HRQoL Issues

To investigate treatment-specific HRQoL issues per sarcoma location, the research team, advised by sarcoma experts, clinicians and sarcoma patients, combined different questions from multiple cancer-specific questionnaires in the EORTC Item Library, a database of items used in fully and partially validated EORTC quality of life questionnaires [41]. This questionnaire included questions about consequences of surgery of the upper and lower extremities (e.g., effects on different activities), surgery of the abdomen (e.g., effects of a stoma), surgery of the uterus, cervix or ovaries (e.g., unfulfilled desire for children), surgery of the head and neck (e.g., swallowing problems), surgery of the breast (e.g., problems seeing yourself naked), chemotherapy (e.g., tingling or numbness in hands or feet), RT (e.g., skin problems), and chemoradiation. All questions focused on long-term side effects or limitations experienced by the sarcoma survivor in the last week. The questions about surgery of the upper and lower extremities were derived from the Toronto Extremity Salvage Score (TESS), a physical disability measure developed specifically for patients undergoing surgery for extremity tumors [42] and were scored on a 6-point scale ranging from 1 ‘impossible’ to 5 ‘no problem at all’ and 6 ‘not applicable’. If a TESS question was answered with ‘impossible’, ‘very difficult’, ‘difficult’ or ‘a little difficult’, the answer to the task was categorized as ‘difficult’. If a TESS question was answered with ‘no problem at all’, the answer to the task was categorized as ‘not difficult’. The questions about side effects and complaints for the other locations were scored on a 4-point scale ranging from 1 ‘not at all’ to 4 ‘very much’. If a question was answered with ‘very much’, ‘quite much’ or ‘a little’, a complaint/side effect was categorized as ‘present’, if the answer was ’not at all’ then it was categorized as ‘not present’. Questions about amputation, stoma, and breast reconstruction were scored as yes/no. Questions about chemotherapy and RT were own design questions and were also scored as yes/no. Only the questions applicable to the survivors’ own reported treatment had to be completed.

### 4.6. Statistical Analysis

Descriptive analyses were used to describe the study population. Categorical variables were presented as numbers and percentages. Normally distributed continuous variables were reported as means and standard deviations, whereas not normally distributed continuous variables were presented with median and interquartile range (IQR). The chi-square or the Fisher Exact Test (for categorical variables with respectively >5 and <5 expected numbers in cells) were used to investigate whether there were significant differences in baseline patient characteristics between the different locations. Mean HRQoL scores of the EORTC-QLQC30 were described per sarcoma location. One-way ANOVA with post hoc Bonferroni analysis was performed to investigate differences in HRQoL scores between the different sarcoma locations. In addition, to adjust for possible confounders, multivariable linear regression analyses with a priori determined potential confounders were performed for location (independent variable) and the separate HRQoL domains (dependent variable). Because of the categorical character of the independent variable, location was transformed into dummy variables with axial skeleton as reference category. In addition, also multivariable linear regression analyses with other reference groups were performed to adjust for confounders in case of significant differences in HRQoL between other sarcoma locations than axial skeleton. Only locations with significant differences in the one-way ANOVA were used as reference groups in the multivariable linear regression. The following a priori determined possible confounders were added in the multivariable linear regression model: age, gender (male vs. female), marital status (married, civil partnership or cohabiting vs. single, widowed or divorced), education (no education, primary or secondary school vs. vocational, college or university), time since diagnosis, sarcoma subtype (STS vs. BS), stage at diagnosis (stage I, II, III, IV), treatment (surgery vs. surgery and RT vs. surgery and chemotherapy vs. surgery, RT and chemotherapy), and comorbidity (0, 1, ≥2 comorbidities). The categorical variables stage at diagnosis, treatment, and comorbidities were transformed into dummy variables, with, respectively, stage I, only surgery and no comorbidities as reference group. To investigate differences in HRQoL between STS and BS, separate independent t-tests were performed for head and neck, chest, and upper and lower extremity sarcomas with sarcoma subtype (STS vs. BS) as independent variable and the separate HRQoL domains as dependent variable. A *p*-value < 0.05 was regarded as statistically significant. Clinically relevant differences between HRQoL scores were determined by the EORTC guidelines as large, medium, small or trivial [20]. Each domain of HRQoL has its own threshold between size classes. The occurrence of treatment-specific HRQoL issues was analyzed in a descriptive way. All analyses were conducted using the Statistical Package for Social Sciences (SPSS) version 22.0 (IBM).

## 5. Conclusions

Different patterns of HRQoL were seen for the different sarcoma locations and a high level of functional disabilities, and symptoms were shown for the specific treatments per sarcoma location. Although generic and cancer-specific HRQoL instruments can provide some information on the HRQoL issues sarcoma patients are dealing with, they are not optimal to cover the heterogeneity of HRQoL issues in the different sarcoma locations. Therefore, a more flexible, comprehensive HRQoL measurement strategy is needed.

## Figures and Tables

**Table 1:** Baseline patient characteristics.

Patient Characteristics	Total *N* = 1099	H and N ^1^ *N* = 72	Thorax *N* = 97	Abd. and R.P. ^2^ *N* = 106	Pelvis and Urogen. Org. ^3^ *N* = 28	Axial Skel. ^4^ *N* = 57	Upper Extr. ^5^ *N* = 116	Lower Extr. ^5^ *N* = 405	Breast *N* = 25	Skin *N* = 110	Other *N* = 83
Gender, N (%), *p* < 0.001											
Male	596 (54)	41 (57)	52 (54)	57 (54)	8 (29)	35 (61)	76 (66)	218 (54)	1 (4)	64 (58)	44 (53)
Female	503 (46)	31 (43)	45 (46)	49 (46)	20 (71)	22 (39)	40 (34)	187 (46)	24 (96)	46 (42)	39 (47)
Age, Mean ± SD, *p* = 0.067											
(In years)	60 ± 15	56 ± 15	60 ± 16	63 ± 13	63 ± 10	60 ± 17	62 ± 16	60 ± 15	66 ± 12	59 ± 16	59 ± 16
Sarcoma Subtype *, N (%), *p* < 0.001											
STS ^6^	829 (75)	35 (49)	59 (61)	102 (96)	28 (100)	1 (2) ^18^	85 (73)	304 (75)	25 (100)	110 (100)	80 (96)
BS ^7^	270 (25)	37 (51)	38 (39)	4 (4) ^17^	0 (0)	56 (98)	31 (27)	101 (25)	0 (0)	0 (0)	3 (4)
Stage, N (%), *p* < 0.001											
I	486 (44)	24 (34)	20 (21)	52 (49)	4 (14)	20 (35)	50 (43)	176 (43)	4 (16)	95 (86)	41 (50)
II	315 (29)	26 (36)	42 (43)	20 (19)	1 (4)	18 (32)	41 (35)	142 (35)	3 (12)	4 (4)	18 (22)
III	134 (12)	1 (1)	19 (20)	21 (20)	0 (0)	0 (0)	17 (15)	60 (15)	3 (12)	1 (1)	12 (14)
IV	58 (5)	8 (11)	2 (2)	7 (6)	2 (7)	12 (20)	5 (4)	14 (4)	2 (8)	1 (1)	5 (6)
Missing	106 (10)	13 (18)	14 (14)	6 (6)	21 (75)	7 (13)	3 (3)	13 (3)	13 (52)	9 (8)	7 (8)
Treatment, N (%), *p* < 0.000											
S ^8^	448 (41)	15 (21)	41 (43)	47 (44)	21 (75)	21 (36)	40 (34)	131 (33)	11 (44)	94 (86)	27 (33)
S and R ^9^	422 (38)	29 (40)	32 (33)	26 (25)	4 (14)	18 (32)	54 (47)	195 (48)	8 (32)	10 (9)	46 (55)
S and C ^10^	79 (7)	9 (13)	9 (9)	12 (11)	0 (0)	3 (5)	9 (8)	33 (8)	2 (8)	1 (1)	1 (1)
S, R and C ^11^	86 (8)	8 (11)	11 (11)	12 (11)	2 (7)	5 (9)	8 (7)	33 (8)	1 (4)	0 (0)	6 (7)
Missing	64 (6)	11 (15)	4 (4)	9 (9)	1 (4)	10 (18)	5 (4)	13 (3)	3 (12)	3 (4)	3 (4)
Time since Diagnosis, Mean ± SD, *p* = 0.223											
(in months)	67 ± 30	68 ± 30	64 ± 31	61 ± 26	67 ± 28	73 ± 30	69 ± 34	69 ± 31	58 ± 22	67 ± 30	67 ± 30
Comorbidities, N (%), *p* = 0.050											
0	369 (34)	30 (42)	28 (29)	29 (28)	5 (18)	18 (32)	31 (27)	159 (40)	4 (16)	38 (34)	27 (32)
1	355 (32)	17 (23)	35 (36)	30 (28)	8 (28)	23 (40)	43 (37)	123 (30)	11 (44)	37 (34)	28 (34)
≥2	375 (34)	25 (35)	34 (35)	47 (44)	15 (54)	16 (28)	42 (36)	123 (30)	10 (40)	35 (32)	28 (34)
Marital Status, N (%), *p* = 0.006											
Together ^12^	857 (78)	55 (76)	68 (70)	81 (76)	25 (89)	42 (74)	87 (75)	335 (83)	21 (84)	90 (82)	53 (64)
Single ^13^	240 (22)	17 (24)	29 (30)	25 (24)	3 (11)	15 (26)	27 (23)	70 (17)	4 (16)	20 (18)	30 (36)
Missing	2 (0)	0 (0)	0 (0)	0 (0)	0 (0)	0 (0)	2 (2)	0 (0)	0 (0)	0 (0)	0 (0)
Education, N (%), *p* = 0.174											
No education ^14^	242 (22)	8 (11)	23 (24)	23 (22)	8 (29)	17 (30)	29 (25)	90 (22)	9 (36)	20 (18)	15 (18)
College ^15^	851 (77)	63 (88)	74 (76)	82 (77)	20 (71)	40 (70)	86 (74)	313 (77)	16 (64)	90 (82)	67 (81)
Missing	6 (1)	1 (1)	0 (0)	1 (1)	0 (0)	0 (0)	1 (1)	2 (1)	0 (0)	0 (0)	1 (1)
Employment, N (%), *p* = 0.115											
Work ^16^	548 (50)	40 (56)	43 (44)	46 (43)	14 (50)	28 (49)	53 (46)	205 (51)	9 (36)	62 (56)	48 (58)
Other	510 (46)	26 (36)	52 (54)	56 (53)	14 (50)	26 (46)	55 (47)	191 (47)	14 (56)	43 (39)	33 (40)
Missing	41 (4)	6 (8)	2 (2)	4 (4)	0 (0)	3 (5)	8 (7)	9 (2)	2 (8)	5 (5)	2 (2)

* Fisher exact not possible for tumor subtype; ^1^ H and N: Head and Neck; ^2^ Abd. and R.P: Abdominal including retroperitoneal; ^3^ Pelvis and urogen. org: Pelvis including urogenital organs; ^4^ Axial skel: Axial skeleton; ^5^ Extr: Extremities; ^6^ STS: Soft Tissue Sarcoma; ^7^ BS: Bone Sarcoma; ^8^ S: Surgery; ^9^ S andR: Surgery and radiotherapy; ^10^ S and C: Surgery and chemotherapy; ^11^ S, R and C: Surgery, radiotherapy and chemotherapy; ^12^ Married, civil partnership or cohabiting; ^13^ Single, widowed or divorced; ^14^ No education, primary, or secondary school; ^15^ Vocational, college, or university; ^16^ Employed or partially disabled; ^17^ Extra-skeletal osteosarcoma in the lower abdomen, Ewing sarcoma of the lower abdomen, Ewing sarcoma retroperitoneal, Ewing sarcoma of the kidney, categorized as BS based on histology; ^18^ Epithelioid angiosarcoma os ilium.

**Table 2:** Results of the one-way ANOVA with Bonferroni and multivariate analyses with axial skeleton as reference group.

EORTC QLQ-C30 Scale per Location	One-Way ANOVA with Post Hoc Bonferroni			Adjusted-Multivariate Linear Regression (Axial Skeleton as Reference Group)			
Column Name	MD	95% CI		MD	95% CI		Clinical Relevance ^1^
Global health status							
Head and Neck[u10] [i.11]	6.9	−3.7	17.5	5.7	−0.6	12.1	N.A.
Chest	12.1	2.2	21.9	11.8	5.7	17.9	Medium
Abd. and R.P.^*^	9.2	−0.5	18.9	9.3	2.9	15.8	N.A.
Pelvis and urogenital organs	7.3	−6.1	20.7	5.2	−3.5	14.0	N.A.
Upper extremities	12.8	3.2	22.4	12.3	6.3	18.4	Medium
Lower extremities	12.0	3.7	20.4	10.1	4.7	15.5	Medium
Breast	9.7	−4.9	24.3	8.2	−1.0	17.3	N.A.
Skin	18.1	8.4	27.7	14.2	7.6	20.8	Medium
Other locations	9.8	−0.3	20.0	8.6	2.1	15.2	N.A.
Physical functioning							
Head and Neck	18.0	5.9	30.1	13.6	6.6	20.5	Small
Chest	22.6	11.3	33.8	18.9	12.2	25.5	Medium
Abd. and R.P.^*^	16.0	5.0	27.0	10.1	3.0	17.1	Small
Pelvis and urogenital organs	21.5	6.2	36.8	13.8	4.2	23.3	Small
Upper extremities	20.8	9.9	31.7	15.7	9.1	22.3	Medium
Lower extremities	15.1	5.6	24.6	8.9	2.9	14.8	Small
Breast	17.1	0.5	33.7	10.9	0.9	21.0	Small
Skin	26.6	15.6	37.6	14.4	7.2	21.6	Medium
Other locations	14.0	2.4	25.5	5.2	−1.9	12.4	N.A.
Role functioning							
Head and Neck	13.6	−2.2	29.3	7.9	−1.5	17.4	N.A.
Chest	18.9	4.3	33.4	12.8	3.7	21.8	Small
Abd. and R.P.*	15.1	0.7	29.5	5.7	−3.9	15.3	N.A.
Pelvis and urogenital organs	25.2	5.3	45.0	11.0	−2.0	24.0	N.A.
Upper extremities	17.3	3.1	31.5	10.1	1.1	19.0	Small
Lower extremities	18.0	5.6	30.3	9.2	1.1	17.3	Small
Breast	10.4	−11.2	32.0	−1.7	−15.3	12.0	N.A.
Skin	26.0	11.8	40.3	9.9	0.1	19.8	Small
Other locations	10.4	−4.7	25.4	−1.1	−10.9	8.6	N.A.
Emotional functioning							
Head and Neck	0.04	−11.9	12.0	1.5	−5.7	8.7	N.A.
Chest	6.5	−4.6	17.6	8.1	1.2	15.0	N.A.
Abd. and R.P.*	4.8	−6.1	15.8	6.8	−0.5	14.1	N.A.
Pelvis and urogenital organs	2.9	−12.2	18.0	4.1	−5.8	13.9	N.A.
Upper extremities	8.5	−2.3	19.3	9.3	2.4	16.1	N.A.
Lower extremities	6.2	−3.2	15.6	6.5	0.3	12.6	N.A.
Breast	−1.7	−18.1	14.7	−0.5	−10.9	9.9	N.A.
Skin	7.3	−3.5	18.2	6.3	−1.2	13.8	N.A.
Other locations	3.5	−7.8	14.9	4.7	−2.7	12.1	N.A.
Cognitive functioning							
Head and Neck	−3.5	−15.3	8.3	−3.0	−10.0	4.1	N.A.
Chest	5.4	−5.5	16.3	4.7	−2.1	11.5	N.A.
Abd. and R.P.*	4.8	−6.0	15.6	3.4	−3.8	10.6	N.A.
Pelvis and urogenital organs	−2.3	−17.2	12.6	−5.1	−14.8	4.6	N.A.
Upper extremities	6.6	−4.0	17.3	5.0	−1.7	11.8	N.A.
Lower extremities	7.2	−2.1	16.5	4.7	−1.3	10.8	N.A.
Breast	4.1	−12.1	20.3	1.3	−8.9	11.5	N.A.
Skin	6.1	−4.6	16.8	−0.3	−7.6	7.1	N.A.
Other locations	2.3	−9.0	13.5	0.7	−6.6	8.0	N.A.
Social functioning							
Head and Neck	5.4	−7.4	18.2	4.4	−3.5	12.3	N.A.
Chest	9.9	−2.0	21.8	7.7	0.1	15.3	N.A.
Abd. and R.P.*	9.4	−2.3	21.2	6.1	−1.9	14.2	N.A.
Pelvis and urogenital organs	6.2	−10.0	22.4	0.9	−9.9	11.8	N.A.
Upper extremities	11.7	0.1	23.3	8.7	1.2	16.3	Small
Lower extremities	10.2	0.1	20.3	7.0	0.2	13.8	Small
Breast	6.2	−11.4	23.8	1.9	−9.5	13.3	N.A.
Skin	14.8	3.2	26.5	7.2	−1.0	15.5	N.A.
Other locations	6.2	−6.1	18.4	2.1	−6.0	10.3	N.A.
Fatigue							
Head and Neck	−7.2	−21.3	6.9	−5.7	−14.0	2.6	N.A.
Chest	−14.8	−27.8	−1.7	−12.0	−19.9	−4.0	Small
Abd. and R.P.*	−7.9	−20.8	5.0	−4.6	−13.1	3.8	N.A.
Pelvis and urogenital organs	−9.1	−26.9	8.6	−2.1	−13.5	9.3	N.A.
Upper extremities	−14.0	−26.7	−1.2	−10.1	−18.0	−2.2	Small
Lower extremities	−14.5	−25.7	−3.4	−9.7	−16.8	−2.6	Small
Breast	−2.6	−21.9	16.7	2.5	−9.5	14.5	N.A.
Skin	−20.4	−33.2	−7.7	−10.5	−19.1	−1.8	Small
Other locations	−5.9	−19.3	7.6	−1.1	−9.7	7.5	N.A.
Nausea/vomiting							
Head and Neck	−2.7	−8.1	2.6	−3.2	−6.5	0.1	N.A.
Chest	−4.0	−8.9	1.0	−4.7	−7.9	−1.5	N.A.
Abd. and R.P.*	0.5	−4.4	5.4	−0.6	−4.0	2.8	N.A.
Pelvis and urogenital organs	−4.4	−11.1	2.4	−5.5	−10.1	−1.0	N.A.
Upper extremities	−3.9	−8.7	0.9	−4.5	−7.7	−1.3	N.A.
Lower extremities	−3.5	−7.7	0.7	−4.0	−6.8	−1.1	N.A.
Breast	−0.3	−7.6	7.1	−1.8	−6.6	3.1	N.A.
Skin	−3.4	−8.2	1.5	−3.2	−6.7	0.2	N.A.
Other locations	−3.1	−8.2	2.0	−3.7	−7.1	−0.3	N.A.
Pain							
Head and Neck	−19.5	−34.1	−4.8	−17.5	−26.3	−8.7	Medium
Chest	−20.9	−34.5	−7.3	−18.0	−26.4	−9.6	Medium
Abd. and R.P.*	−21.2	−34.6	−7.8	−17.0	−25.9	−8.1	Medium
Pelvis and urogenital organs	−19.6	−38.1	−1.1	−12.4	−24.4	−0.4	Small
Upper extremities	−24.8	−38.1	−11.6	−21.5	−29.9	−13.1	Large
Lower extremities	−19.9	−31.5	−8.3	−16.1	−23.6	−8.5	Medium
Breast	−17.7	−37.8	2.4	−13.0	−25.6	−0.3	N.A.
Skin	−27.4	−40.7	−14.0	−17.5	−26.7	−8.4	Medium
Other locations	−17.5	−31.4	−3.5	−12.9	−22.0	−3.9	Small
Dyspnea							
Head and Neck	−6.2	−19.2	6.8	−5.7	−13.5	2.1	N.A.
Chest	−5.0	−17.1	7.0	−5.1	−12.6	2.4	N.A.
Abd. and R.P.*	−5.5	−17.5	6.4	−6.5	−14.4	1.5	N.A.
Pelvis and urogenital organs	−4.2	−20.7	12.2	−3.5	−14.2	7.2	N.A.
Upper extremities	−8.7	−20.5	3.0	−9.1	−16.5	−1.7	N.A.
Lower extremities	−9.0	−19.3	1.2	−7.6	−14.3	−1.0	N.A.
Breast	2.7	−15.2	20.6	3.6	−7.6	14.9	N.A.
Skin	−10.3	−22.2	1.5	−6.3	−14.4	1.8	N.A.
Other locations	−8.1	−20.6	4.3	−6.6	−14.6	1.5	N.A.
Insomnia							
Head and Neck	−16.1	−32.8	0.6	−15.7	−25.8	−5.6	N.A.
Chest	−14.7	−30.2	0.8	−15.2	−24.9	−5.5	N.A.
Abd. and R.P.*	−13.7	−28.9	1.6	−14.0	−24.3	−3.8	N.A.
Pelvis and urogenital organs	−16.7	−37.8	4.4	−17.4	−31.3	−3.5	N.A.
Upper extremities	−16.7	−31.8	−1.6	−15.5	−25.1	−5.8	Medium
Lower extremities	−15.6	−28.7	−2.4	−13.9	−22.5	−5.2	Medium
Breast	−11.8	−34.8	11.1	−15.2	−29.8	−0.6	N.A.
Skin	−22.9	−38.1	−7.8	−17.7	−28.2	−7.1	Medium
Other locations	−13.3	−29.3	2.7	−11.6	−22.0	−1.1	N.A.
Appetite Loss							
Head and Neck	−4.1	−13.9	5.6	−3.8	−9.8	2.2	N.A.
Chest	−6.3	−15.4	2.7	−6.7	−12.5	−0.9	N.A.
Abd. and R.P.*	−2.9	−11.8	6.0	−3.2	−9.3	3.0	N.A.
Pelvis and urogenital organs	−7.6	−19.9	4.7	−9.6	−17.9	−1.3	N.A.
Upper extremities	−8.7	−17.5	0.1	−8.2	−13.9	−2.5	N.A.
Lower extremities	−9.4	−17.1	−1.7	−8.6	−13.7	−3.4	Small
Breast	−1.7	−15.1	11.7	−3.3	−12.0	5.4	N.A.
Skin	−8.6	−17.4	0.3	−6.3	−12.5	−0.01	N.A.
Other locations	−7.3	−16.7	2.0	−7.1	−13.3	−0.9	N.A.
Constipation							
Head and Neck	−3.3	−15.0	8.4	−2.8	−9.8	4.3	N.A.
Chest	−9.1	−19.9	1.8	−8.1	−14.9	−1.3	N.A.
Abd. and R.P. *	−1.4	−12.0	9.3	−0.2	−7.4	7.0	N.A.
Pelvis and urogenital organs	−0.8	−15.5	13.9	−2.0	−11.7	7.7	N.A.
Upper extremities	−7.4	−18.0	3.2	−5.6	−12.4	1.1	N.A.
Lower extremities	−8.1	−17.3	1.1	−6.1	−12.2	−0.1	N.A.
Breast	−1.5	−17.4	14.5	−2.7	−12.9	7.6	N.A.
Skin	−11.9	−22.5	−1.3	−7.1	−14.5	0.2	N.A.
Other locations	−7.3	−18.4	3.9	−5.2	−12.5	2.1	N.A.
Diarrhea							
Head and Neck	2.7	−6.4	11.8	3.4	−2.1	9.0	N.A.
Chest	−2.0	−10.4	6.5	−1.8	−7.2	3.5	N.A.
Abd. and R.P.*	6.0	−2.3	14.3	5.7	−0.0	11.4	N.A.
Pelvis and urogenital organs	5.0	−6.4	16.5	5.9	−1.7	13.6	N.A.
Upper extremities	−0.5	−8.7	7.7	0.008	−5.3	5.4	N.A.
Lower extremities	−0.5	−7.7	6.7	0.3	−4.5	5.1	N.A.
Breast	0.1	−12.3	12.4	0.3	−7.8	8.3	N.A.
Skin	−1.9	−10.2	6.3	0.1	−5.7	5.9	N.A.
Other locations	2.7	−6.0	11.4	3.6	−2.2	9.4	N.A.
Financial							
Head and Neck	0.5	−12.6	13.6	0.5	−7.5	8.6	N.A.
Chest	−10.1	−22.3	2.0	−8.9	−16.6	−1.2	N.A.
Abd. and R.P.*	−7.6	−19.6	4.4	−5.2	−13.3	3.0	N.A.
Pelvis and urogenital organs	−4.7	−21.3	11.8	−1.4	−12.4	9.6	N.A.
Upper extremities	−9.0	−20.8	2.9	−7.0	−14.6	0.7	N.A.
Lower extremities	−6.3	−16.7	4.0	−3.8	−10.7	3.1	N.A.
Breast	−0.3	−18.3	17.7	4.3	−7.3	15.9	N.A.
Skin	−10.7	−22.6	1.2	−5.9	−14.3	2.4	N.A.
Other locations	−3.5	−16.0	9.0	−1.1	−9.3	7.2	N.A.

^1^ Clinical relevance is only mentioned for the locations that were significant in the one-way ANOVA and remained significant after adjustment for confounders in the multivariate analyses [20]. N.A. = Not applicable. * Abd. and R.P. = Abdominal including retroperitoneal.

## Supplementary Materials

The following are available online at https://www.mdpi.com/2072-6694/12/11/3083/s1, Table S1: Results of post hoc analyses and multivariate analyses with skin, head and neck, and abdominal including retroperitoneal sarcomas as reference group, Table S2: treatment-specific HRQoL issues after surgery of the upper extremities, Table S3: treatment-specific HRQoL issues after surgery of the lower extremities, Table S4: treatment-specific HRQoL issues after surgery in the abdomen, Table S5: treatment-specific HRQoL issues after surgery of the cervix, uterus or ovaries, Table S6: treatment-specific HRQoL issues after surgery in the head or neck area, Table S7: treatment-specific HRQoL issues after surgery of the breast, Table S8: treatment-specific HRQoL issues after chemotherapy, radiotherapy, and chemoradiation, Text S1: Overview of the specific sarcomas per location category.
