# Supplementary material for: Unraveling the Heterogeneity of Sarcoma Survivors’ Health-Related Quality of Life Regarding Primary Sarcoma Location: Results from the SURVSARC Study
Source: Cancers (Basel). 2020 Oct 22;12(11):3083. doi: 10.3390/cancers12113083 (PMC7690571; doi:10.3390/cancers12113083)
Supplement: Supplementary file 1 [file cancers-12-03083-s001.pdf]

cancers

MDPI

Article

Unravelling the Heterogeneity of Sarcoma Survivors' Health-Related Quality of Life Regarding Primary Sarcoma Location: Results from the SURVSARC Study

Ilse van Eck, Dide den Hollander, Ingrid M.E. Desar, Vicky L.M.N. Soomers, Michiel A.J. van de Sande, Jacco J. de Haan, Cornelis Verhoef, Ingeborg J.H. Vriens, Johannes J. Bonenkamp, Winette T.A. van der Graaf, Winan J. van Houdt and Olga Husson

Supplementary Materials

Text S1: Overview of the specific Sarcomas per Location Category

Category 1: Head & Neck

- C05.1 Soft palate.
- C07.0 Malignant neoplasm of parotid gland.
- C08.0 Submandibular gland.
- C30.0 Nasal cavity.
- C31.1 Ethmoidal sinus.
- C31.8 Overlapping lesion of accessory sinuses.
- C32.3 Laryngeal Cartilage
- C41.0 Bones of skull and face
- C41.1 Mandible
- C49.0 Connective and soft tissue of head, face and neck
- C69.6 Orbit, unspecified
- C76.0 Nose, unspecified

Category 2: Chest

- C34.1 Upper lobe, bronchus or lung
- C34.3 Lower Lobe, bronchus or lung
- C34.9 Bronchus or lung, unspecified
- C38.2 Posterior mediastinum
- C38.3 Mediastinum, part unspecified
- C41.3 Ribs, sternum and clavicle
- C49.3 Connective and soft tissue of thorax
- C49.6 Connective and soft tissue of trunk, unspecified

Category 3: Abdominal including retroperitoneal

- C16.3 Stomach + pyloric antrum
- C22.0 Liver
- C48.0 Retroperitoneum
- C48.1 Specified parts of peritoneum
- C49.4 Connective and soft tissue of abdomen
- C64.0 Kidney, unspecified

- C66.0 Ureter

Category 4: Pelvis including urogenital organs

- C20.0 Rectum, unspecified
- C51.0 Labium majus
- C51.9 Vulva, unspecified
- C53.9 Cervix uteri, unspecified
- C54.1 Corpus Uteri: Endometrium
- C54.2 Corpus Uteri: Myometrium
- C54.9 Corpus uteri, unspecified
- C56.0 Ovary
- C63.1 Spermatic cord
- C63.2 Scrotum
- C63.7 Other specified male genital organs: Seminal vesicle, Tunica vaginalis
- C67.9 Bladder, unspecified

Category 5: Axial Skeleton

- C41.2 Vertebral column
- C41.4 Pelvic bones, sacrum and coccyx

Category 6: Upper extremities

- C40.0 Scapula and long bones of upper limb
- C40.1 Short bones of upper limb
- C49.1 Connective and soft tissue of upper limb, including shoulder

Category 7: Lower extremities

- C40.2 Long bones of lower limb
- C40.3 Short bones of lower limb
- C49.2 Connective and soft tissue of lower limb, including hip

Category 8: Breast

- C50.1 Central portion of breast
- C50.2 Upper-inner quadrant of breast
- C50.3 Lower-inner quadrant of breast
- C50.4 Upper-outer quadrant of breast
- C50.5 Lower-outer quadrant of breast
- C50.6 Axillary tail of breast
- C50.8 Overlapping lesion of breast
- C50.9 Breast, unspecified

Category 9: Skin

- C44.1 Skin of eyelid, including canthus
- C44.3 Skin of other and unspecified parts of face
- C44.4 Skin of scalp and neck
- C44.5 Skin of trunk
- C44.6 Skin of upper limb, including shoulder
- C44.7 Skin of lower limb, including hip

Category 10: Other:

- C47.0 Peripheral nerves of head, face and neck

- C47.1 Peripheral nerves of upper limb, including shoulder
- C47.2 Peripheral nerves of lower limb, including hip
- C47.3 Peripheral nerves of thorax
- C47.4 Peripheral nerves of abdomen
- C47.5 Peripheral nerves of pelvis
- C47.6 Peripheral nerves of trunk, unspecified
- C49.5 Connective and soft tissue of pelvis
- C49.9 Connective and soft tissue, unspecified
- C70.0 Cerebral meninges
- C80.9 Malignant neoplasm, primary site unspecified

Table S1. Results of Post-hoc analyses and multivariate analyses with skin, head & neck and abdominal including retroperitoneal sarcomas as reference group.

	**One-way ANOVA with Post Hoc Bonferroni**			**Adjusted - Multi-variate linear regression**			
	**MD**	**95% CI**		**MD**	**95% CI**		**Clinical relevance [1]**
Global health status							
Compared to skin							
Head & Neck	−11.2	−20.3	−2.1	−8.5 [a]	−14.4	−2.3	Small
Abd. + R.P.*	−8.9	−16.9	−0.9	−4.9 [a]	−9.7	−0.1	Small
Physical functioning							
Compared to skin							
Abd. + R.P.*	−10.6	−19.7	−1.5	−4.3	−9.6	0.9	N.A.
Lower extremities	−11.5	−18.6	−4.3	−5.5 [a]	−9.9	−1.2	Small
Other locations	−12.6	−22.3	−2.9	−9.2 [a]	−14.8	−3.6	Small
Role functioning							
Compared to skin							
Other locations	−15.7	−28.3	−3.0	−11.0 [a]	−18.7	−3.4	Small
Cognitive functioning							
*Compared to H&N***							
Upper extremities	10.2	0.1	20.2	8.0 [b]	2.0	14.0	Small
Lower extremities	10.7	2.1	19.3	7.7 [b]	2.6	12.9	Small
Fatigue							
Compared to Skin							
Head & Neck	13.2	1.3	25.2	4.8 [a]	−2.9	12.4	N.A.
Abd. + R.P.*	12.5	2.0	23.1	5.9 [a]	−0.4	12.1	N.A.
Breast	17.9	0.05	35.7	13.0 [a]	2.3	23.6	Small
Other locations	14.6	3.3	25.8	9.4 [a]	2.6	16.1	Small
Nausea/vomiting							
*Compared to Abd. + R.P.**							
Chest	−4.5	−8.6	−0.3	−4.1 [c]	−6.7	−1.5	Small
Upper extremities	−4.4	−8.4	−0.4	−3.9 [c]	−6.4	−1.4	Small
Lower extremities	−4.0	−7.2	−0.8	−3.4 [c]	−5.4	−1.3	Small
Appetite Loss							
*Compared to Abd. + R.P.**							
Lower extremities	−6.5	−12.4	−0.7	−5.4 [c]	−9.1	−1.8	Small
Constipation			¤				
*Compared to Abd. + R.P.**							
Skin	−10.6	−19.3	−1.8	−7.0 [c]	−12.3	−1.6	Small
Diarrhoea							
*Compared to Abd. + R.P.**							
Chest	−7.9	−14.9	−1.0	−7.5 [c]	−11.8	−3.2	Medium

Lower extremities	−6.5	−11.9	−1.1	−5.4 [c]	−8.8	−2.0	Small
Skin	−7.9	−14.7	−1.1	−5.6 [c]	−9.8	−1.4	Small

Only locations with significant differences in the one-way ANOVA were used as reference groups in the multivariable linear regression. [1] Clinical relevance is only mentioned for the locations that were significant in the One-way ANOVA and remained significant after adjustment for confounders in the multivariate analyses [26]. N.A. = Not Applicable; * Abd. + R.P. = abdominal including retroperitoneal; ** H&N = Head & Neck. [a] Skin sarcomas as reference group; [b] Head & Neck sarcomas as reference group; [c] Abdominal including retroperitoneal sarcomas as reference group.

Table S2. Treatment specific HRQoL issues after surgery of the upper extremities.

N = 116	Difficult Total N (%)	Difficult for amputation N (%)	Difficult for no amputation N (%)	Not difficult N (%)	Not applicable N (%)	Missings N (%)
Putting on a pair of trousers	15 (13)	7/17 (41)	8/65 (12)	59 (51)	7 (6)	35 (30)
Tying shoe laces	23 (20)	12/17 (71)	11/65 (17)	51 (44)	7 (6)	35 (30)
Putting on a pair of socks/stockings	14 (12)	7/17 (41)	7/65 (11)	60 (52)	7 (6)	35 (30)
Showering	14 (12)	5/17 (29)	9/65 (14)	61 (53)	6 (5)	35 (30)
Dressing the arms and upper body	22 (19)	6/17 (35)	16/65 (25)	54 (47)	5 (4)	35 (30)
Buttoning a shirt	26 (23)	12/17 (71)	14/65 (22)	49 (42)	6 (5)	35 (30)
Tying a tie/bow at the neck of a blouse	19 (16)	9/17 (53)	10/65 (15)	46 (40)	16 (14)	35 (30)
Putting on make-up or shaving	17 (15)	4/17 (24)	13/65 (20)	58 (50)	6 (5)	35 (30)
Brushing teeth	10 (9)	4/17 (24)	6/65 (9)	64 (55)	6 (5)	36 (31)
Brushing hair	13 (11)	2/17 (12)	11/65 (17)	60 (52)	8 (7)	35 (30)
Performing light household chores	22 (19)	8/17 (47)	14/65 (22)	50 (43)	9 (8)	35 (30)
Gardening	37 (32)	13/17 (76)	24/65 (37)	27 (23)	16 (14)	36 (31)
Preparing and serving meals	26 (22)	12/17 (71)	14/65 (22)	46 (40)	9 (8)	35 (30)
Cutting food while eating	27 (23)	14/17 (82)	13/65 (20)	47 (41)	6 (5)	36 (31)
Drinking from a glass	8 (7)	3/17 (18)	5/65 (8)	65 (56)	7 (6)	36 (31)
Performing heavy household chores	46 (39)	14/17 (82)	32/65 (49)	24 (21)	10 (9)	36 (31)
Going shopping	21 (18)	9/17 (53)	12/65 (18)	51 (44)	8 (7)	36 (31)
Giving or receiving change (coins/notes)	25 (22)	11/17 (65)	14/65 (22)	49 (42)	6 (5)	36 (31)
Carrying a shopping bag or briefcase	33 (28)	11/17 (65)	22/65 (34)	40 (35)	7 (6)	36 (31)
Lifting a box to an overhead shelf	43 (37)	14/17 (82)	29/65 (45)	32 (28)	5 (4)	36 (31)
Turning a key in lock	11 (10)	5/17 (29)	6/65 (9)	63 (54)	6 (5)	36 (31)
Pushing or pulling open a door	17 (15)	4/17 (24)	13/65 (20)	56 (48)	7 (6)	36 (31)
Writing	18 (15)	10/17 (59)	8/65 (12)	54 (47)	8 (7)	36 (31)
Picking up small items	17 (15)	7/17 (41)	10/65 (15)	58 (50)	5 (4)	36 (31)
Completing usual duties at work/at home	31 (27)	12/17 (71)	19/65 (29)	42 (36)	7 (6)	36 (31)
Working usual number of hours	21 (18)	7/17 (41)	14/65 (22)	40 (35)	19 (16)	36 (31)
Participating in usual leisure activities	30 (26)	13/17 (76)	17/65 (26)	39 (33)	11 (10)	36 (31)
Socialising with friends and family	9 (8)	4/17 (24)	5/65 (8)	63 (54)	8 (7)	36 (31)
Participating in usual sporting activities	37 (32)	14/17 (82)	23/65 (35)	34 (29)	9 (8)	36 (31)

Table S3. Treatment specific HRQoL issues after surgery of the lower extremities.

N = 405	Difficult Total N (%)	Difficult for amputation N (%)	Difficult for no amputation N (%)	Not difficult N (%)	Not applicable N (%)	Missings N (%)

Putting on a pair of trousers	103 (25)	19/37 (51)	84/317 (26)	231 (57)	14 (4)	57 (14)
Putting on shoes	114 (28)	21/37 (57)	93/317 (29)	218 (53)	14 (4)	59 (15)
Putting on a pair of socks of stockings	121 (30)	19/37 (51)	102/317 (32)	214 (53)	13 (3)	57 (14)
Showering	66 (16)	26/37 (70)	40/317 (13)	266 (66)	16 (4)	57 (14)
Performing light household chores	70 (18)	20/37 (54)	50/317 (16)	252 (62)	25 (6)	58 (14)
Gardening	150 (37)	28/37 (76)	122/317 (38)	156 (39)	42 (10)	57 (14)
Preparing and serving meals	51 (13)	18/37 (49)	33/317 (10)	267 (66)	29 (7)	58 (14)
Going shopping	123 (31)	28/37 (76)	95/317 (30)	207 (51)	17 (4)	58 (14)
Performing heavy household chores	155 (39)	29/37 (78)	126/317 (40)	170 (42)	22 (5)	58 (14)
Getting in and out of the bath	88 (22)	19/37 (51)	69/317 (22)	181 (44)	73 (18)	63 (16)
Getting out of bed	77 (19)	15/37 (41)	62/317 (20)	254 (62)	14 (4)	60 (15)
Rising from a chair	110 (27)	18/37 (49)	92/317 (29)	222 (54)	14 (4)	59 (15)
Kneeling	228 (56)	32/37 (86)	196/317 (62)	106 (26)	11 (3)	60 (15)
Bending to pick something off the floor	150 (37)	26/37 (70)	124/317 (39)	183 (45)	12 (3)	60 (15)
Walking upstairs	150 (37)	25/37 (68)	125/317 (39)	182 (45)	13 (3)	60 (15)
Walking downstairs	138 (34)	30/37 (81)	108/317 (34)	194 (48)	13 (3)	60 (15)
Driving	53 (13)	11/37 (30)	42/317 (13)	253 (62)	39 (10)	60 (15)
Walking within the house	66 (16)	23/37 (62)	43/317 (14)	266 (66)	13 (3)	60 (15)
Walking outdoors	125 (31)	28/37 (76)	97/317 (31)	206 (51)	13 (3)	61 (15)
Sitting	43 (11)	11/37 (30)	32/317 (10)	288 (71)	13 (3)	61 (15)
Walking up or down hills or a ramp	186 (46)	31/37 (84)	155/317 (49)	145 (36)	13 (3)	61 (15)
Standing	59 (15)	17/37 (46)	42/317 (13)	273 (67)	13 (3)	60 (15)
Getting up from kneeling	233 (57)	29/37 (78)	204/317 (64)	96 (24)	16 (4)	60 (15)
Getting in and out of a car	138 (34)	20/37 (54)	118/317 (37)	193 (48)	13 (3)	61 (15)
Participating in sexual activities	91 (23)	22/37 (59)	69/317 (22)	200 (49)	52 (13)	62 (15)
Completing usual duties at work/at home	129 (32)	23/37 (62)	106/317 (33)	189 (46)	28 (7)	59 (15)
Working usual number of hours	105 (26)	20/37 (54)	85/317 (27)	184 (45)	57 (14)	59 (15)
Participating in usual leisure activities	138 (34)	29/37 (78)	109/317 (34)	190 (47)	18 (4)	59 (15)
Socialising with friends and family	52 (13)	17/37 (46)	35/317 (11)	277 (68)	16 (4)	60 (15)
Participating in usual sporting activities	183 (45)	29/37 (78)	154/317 (49)	133 (33)	29 (7)	60 (15)

Table S4. Treatment specific HRQoL issues after surgery in the abdomen.

	Present N (%)	Not present N (%)	Missings N (%)
Answered by all abdominal including retroperitoneal sarcoma survivors N = 106			
Abdominal pain	34 (32)	56 (53)	16 (15)
Bloated feeling in the abdomen	34 (32)	55 (52)	17 (16)
Problems with eating	15 (14)	74 (70)	17 (16)
Problems with drinking	7 (7)	82 (77)	17 (16)
Stomach complaints	20 (19)	69 (65)	17 (16)
Felt full up too quickly while eating	23 (22)	66 (62)	17 (16)
Worries about weight	38 (36)	52 (49)	16 (15)
Answered by only abdominal including retroperitoneal sarcoma survivors without ostomy N = 99			

Unintentional release of gas/flatulence	38 (39)	45 (45)	16 (16)
Unintentional release of stool	12 (12)	72 (72)	16 (16)
Embarrassed due to defecation pattern	22 (22)	61 (62)	16 (16)
Answered by only abdominal including retroperitoneal sarcoma survivors with ostomy N = 7			
Unintentional release of gas/flatulence from stoma bag	3 (43)	4 (57)	0 (0)
Leakage of stools from stoma bag	2 (29)	5 (71)	0 (0)
Embarrassed because of the stoma	3 (43)	4 (57)	0 (0)
Problems caring for the stoma	1 (14)	6 (86)	0 (0)

Table S5. Treatment specific HRQoL issues after surgery of the cervix, uterus or ovaries.

N = 17	Present N (%)	Not present N (%)	Not applicable N (%)	Missings N (%)
Pain in the back	9 (53)	6 (35)	0 (0)	2 (12)
Hurry to toilet urine urge	7 (41)	8 (47)	0 (0)	2 (12)
Unintentional release of urine	6 (35)	9 (53)	0 (0)	2 (12)
Hurry toilet stool urge	3 (18)	12 (70)	0 (0)	2 (12)
Unintentional release of stool	0 (0)	15 (88)	0 (0)	2 (12)
Flatulence	9 (53)	6 (35)	0 (0)	2 (12)
Abdominal cramps	7 (41)	8 (47)	0 (0)	2 (12)
Bloated feeling in the abdomen	7 (41)	8 (47)	0 (0)	2 (12)
Worries about ability to have children	0 (0)	3 (18)	12 (70)	2 (12)
Complaints similar to menopause	9 (53)	4 (23)	2 (12)	2 (12)

Table S6. Treatment specific HRQoL issues after surgery in the head or neck area.

N = 72	Present N (%)	Not present N (%)	Missings N (%)
Pain in operated area	16 (22)	35 (49)	21 (29)
Sensitive mouth	15 (21)	35 (49)	22 (30)
Problems swallowing liquids	11 (15)	40 (56)	21 (29)
Problems swallowing pureed food	9 (13)	42 (58)	21 (29)
Problems swallowing solid foods	13 (18)	38 (53)	21 (29)
Choking	10 (14)	41 (57)	21 (29)
Problems chewing	18 (25)	33 (46)	21 (29)
Problems with teeth	12 (17)	39 (54)	21 (29)
Dry mouth	24 (33)	27 (38)	21 (29)
Problems with coughing	11 (15)	40 (56)	21 (29)
Problems with hoarseness	10 (14)	41 (57)	21 (29)
Problems with smell	20 (28)	31 (43)	21 (29)
Problems with sense of taste	17 (24)	34 (47)	21 (29)
Trouble eating in front of other people	16 (22)	35 (49)	21 (29)
Problems speaking clearly	25 (35)	26 (36)	21 (29)
Difficulties raising the arm or moving it sideways	7 (10)	44 (61)	21 (29)
Pain in shoulders	14 (19)	37 (52)	21 (29)

Table S7. Treatment specific HRQoL issues after surgery of the breast.

N = 25	Present N (%)	Not present N (%)	Missings N (%)
Breast reconstruction	5 (20)	15 (60)	5 (20)

Swollen arm or hand	4 (16)	16 (64)	5 (20)
Difficulties raising the arm or moving it sideways	8 (32)	12 (48)	5 (20)
Pain in the area of affected breast	7 (28)	13 (52)	5 (20)
Swelling in the area of affected breast	4 (16)	16 (64)	5 (20)
Numbness or tingling in arm or shoulder	4 (16)	16 (64)	5 (20)
Difficulties looking at yourself naked	12 (48)	8 (32)	5 (20)

Table S8. Treatment specific HRQoL issues after chemotherapy, radiotherapy and chemoradiation.

	**Present N (%)**	**Not present N (%)**	**Missings N (%)**
Chemotherapy (n = 86)			
Tingling or numbness in hands or feet	37 (43)	49 (57)	0 (0)
Pain in muscles or joints	33 (38)	53 (62)	0 (0)
Food and drink taste different than usual	19 (22)	67 (78)	0 (0)
Dry mouth	29 (34)	57 (66)	0 (0)
Sensitive mouth	19 (22)	67 (78)	0 (0)
Skin problems (e.g. itchy, dry)	35 (41)	51 (59)	0 (0)
Problems with hair	29 (34)	57 (66)	0 (0)
Concerned about your ability to have children	13 (15)	73 (85)	0 (0)
Radiotherapy (n = 426)			
Skin problems (e.g., itchy, dry)	150 (35)	276 (65)	0 (0)
Muscle cramps at the site of radiation	95 (22)	327 (77)	4 (1)
Scars at site of radiation	196 (45)	227 (54)	3 (1)
Muscle weakness or loss of sensation at site of radiation	201 (47)	223 (53)	2 (0)
Dry cough	50 (12)	372 (87)	4 (1)
Irradiated bones that spontaneously broke	9 (2)	416 (98)	1 (0)
Chemoradiation (n = 98)			
Tingling or numbness in hands or feet	42 (43)	56 (57)	0 (0)
Pain in muscles or joints	39 (40)	59 (60)	0 (0)
Food and drink taste different than usual	41 (42)	57 (58)	0 (0)
Dry mouth	44 (45)	54 (55)	0 (0)
Sensitive mouth	29 (30)	69 (70)	0 (0)
Skin problems (e.g., itchy, dry)	42 (43)	56 (57)	0 (0)
Problems with hair	40 (41)	58 (59)	0 (0)
Concerned about your ability to have children	11 (11)	85 (87)	2 (2)
Scars at site of radiation	30 (31)	65 (66)	3 (3)
Muscle weakness or loss of sensation at site of radiation	43 (44)	54 (55)	1 (1)
Dry cough	18 (18)	79 (81)	1 (1)
Irradiated bones that spontaneously broke	4 (4)	93 (95)	1 (1)

© 2020 by the authors. Licensee MDPI, Basel, Switzerland. This article is an open access article distributed under the terms and conditions of the Creative Commons Attribution (CC BY) license (http://creativecommons.org/licenses/by/4.0/).
